# Dietary Intake of Canadian Middle‐Aged Endurance Athletes

**DOI:** 10.1111/jhn.70182

**Published:** 2025-12-25

**Authors:** B. Ghomeshi, L. McPhee, O. Morello, M. Ismail, M. Kebbe, J. O. Totosy de Zepetnek

**Affiliations:** ^1^ College of Medicine and Public Health Flinders University Bedford Park South Australia Australia; ^2^ Faculty of Kinesiology and Health Studies University of Regina Saskatchewan Canada; ^3^ School of Nutrition Toronto Metropolitan University Toronto Ontario Canada; ^4^ Faculty of Health Studies University of Western Ontario London Ontario Canada; ^5^ Faculty of Kinesiology University of New Brunswick New Brunswick Canada

**Keywords:** dietary intake, dietary reference intakes, endurance athletes, middle‐age, sport nutrition guidelines, supplements

## Abstract

**Introduction:**

The purpose of the present study was to assess dietary intakes of Canadian non‐elite middle‐aged endurance athletes and explore potential sex differences.

**Methods:**

Non‐consecutive 3‐day food intake records (FIR) were analyzed using Cronometer. Intakes were compared to dietary reference intakes (DRIs), acceptable macronutrient distribution ranges (AMDRs), and sports nutrition guidelines when available. Dietary intakes, including the proportion of males versus females meeting recommendations for each nutrient, were assessed using descriptive statistics, independent *t*‐tests, and/or Fisher's exact test.

**Results:**

A total of *n* = 44 (28 M/16 F) middle‐aged endurance athletes completed the study (age = 50.4 ± 7.2 years, BMI = 23.6 ± 3.2 kg/m^2^, moderate‐vigorous activity [MVA] = 627.0 ± 3.2 min/week). All athletes met recommendations for protein (PRO), but not carbohydrates (CHO; 20.5% met sport nutrition guidelines) or fat (FAT; 54.5% exceeded AMDRs), with no differences between males and females. Athletes did not meet the recommended intakes for vitamin D (4.5% [38.6% with supplementation]), vitamin E (34.1% [47.7% with supplementation]), potassium (15.9%), calcium (50.0% [61.4% with supplementation]), or magnesium (65.9% [78.0% with supplementation]), and exceed the recommended intake of sodium (70.5% exceeded DRI). Females were more likely than males to have intakes below the DRIs for several vitamins and minerals, however many of these differences disappeared when supplement use was considered.

**Conclusion:**

Middle‐aged athletes require diets that not only support physical performance but also promote healthy aging. Findings in the present study indicate that Canadian non‐elite middle‐aged endurance athletes are not meeting recommended intakes of CHO, FAT, and many micronutrients; improving adherence to recommended intakes may help these athletes achieve optimal performance, taking into consideration age‐ and sex‐related physiological changes. Future directions should investigate longitudinal dietary intake among middle‐aged endurance athletes, as well as dietary intake based on menopausal stage.

## Summary

- Middle‐aged endurance athletes require diets that not only support physical performance but also promote healthy aging.
- Canadian middle‐aged endurance athletes did not meet the recommended intakes of total kcal, carbohydrates, fat‐soluble vitamins, vitamin B9, potassium, calcium, or magnesium.
- Canadian middle‐aged endurance athletes exceeded the recommended intakes of total fat, saturated fat, and sodium.
- Females were at greater risk than males for not meeting recommended micronutrient intakes, however many of these differences disappeared when supplement use was considered.
- Improving adherence to recommended intakes may help middle‐aged non‐elite endurance athletes achieve optimal performance, taking into consideration age‐ and sex‐related physiological changes.

**Abbreviations**
AI: adequate intake
AMDR: acceptable macronutrient distribution range
CHO: carbohydrate
DRI: dietary reference intake
EER: estimation of energy requirements
FIR: food intake record
IPAQ = SF: International Physical Activity Questionnaire – Short Form
MSJ: Mifflin‐St Jeor equation
MVA: moderate‐vigorous activity
PA: physical activity
PRO: protein
RDA: Recommended Dietary Allowance
UL: upper limit

## 1 Introduction

Compared with non‐athletes, endurance athletes require greater caloric intake (energy expenditure can be up to two to three times higher [1]) and higher amounts of key micronutrients. That is, higher carbohydrate (CHO) intake to fuel increased energy expenditure [2], higher protein (PRO) intake to support training and post‐exercise recovery and adaptation, especially within skeletal muscle [2, 3], and while fat (FAT) intake recommendations are not different from non‐athletes, an emphasis on high‐quality fats—particularly omega‐3 and omega‐6 fatty acids— is important for modulating inflammation, supporting cardiovascular function, and promoting recovery [4, 5, 6]. Further, while no athlete‐specific micronutrient recommendations exist, endurance athletes have elevated needs for fat‐soluble vitamins A, D, and E, B vitamins, vitamin C, and minerals such as calcium, potassium, iron, magnesium, and selenium due to higher metabolic turnover, oxidative stress, and sweat losses [7, 8, 9]. Therefore, consuming sufficient nutrients as well as replenishing energy reserves are key factors for athletic success as well as and overall health [1]. However, previous studies have reported that endurance athletes often have inadequate intakes of total energy, macronutrients, and many micronutrients [10, 11, 12].

Adequate macronutrient and micronutrient intake is particularly critical for middle‐aged endurance athletes, who face age‐related declines in endurance performance and muscle function, along with changes in body composition that reduce glycogen storage capacity, lower resting metabolic rate, decrease metabolic efficiency, and slow recovery from training [13, 14]. Further, sex differences exist in performance and response to exercise training due to fluctuations in sex hormones throughout the menstrual cycle as well as during peri‐ and post‐menopausal periods in females [15, 16], necessitating sex‐specific nutritional recommendations. In addition to physiological differences, males and females exhibit distinct behavioural patterns including males generally aiming to increase muscle mass while females more often reporting attempts at weight loss [17]. Previous work have shown female athletes often consume less than their energy requirements [18] and are less likely to meet recommendations of certain micronutrients (e.g., iron, B_12_, calcium, vitamin D) when compared to males [12, 18, 19, 20]. A recent systematic review examining dietary practices of younger (35–50 years) and older (> 50 years) athletes competing in a variety of sporting disciplines (from endurance to power‐based) reported the highest protein intake among younger male athletes, and higher protein intake among older (vs. younger) female athletes [21]. Further, a study by Graybeal et al. in 2022 reported that older endurance athletes (> 40 years) used dietary supplements (i.e., to help meet micronutrient requirements) more than younger endurance athletes (18–29 years) [22]. Collectively, these findings suggest older athletes, particularly females, may be more intentional in tailoring their dietary strategies to support performance, recovery, and age‐related nutritional needs.

To date, much of the existing literature reporting on dietary intakes are conducted among younger elite athletes, do not consider sex differences, and focus primarily on macronutrients or specific micronutrients. The purpose of the present study was to comprehensively assess the dietary intake of a cohort of Canadian middle‐aged (40–65 years) non‐elite (i.e., not competing at a professional level) endurance athletes (≥ 8 h of moderate‐vigorous aerobic training per week), and to explore potential sex differences.

## 2 Materials and Methods

### 2.1 Participants

Data were collected for the present cross‐sectional study between January and August 2020. Participants were recruited via social media (Facebook) targeting endurance athlete communities across Canada, emails to friends and colleagues, and via snowball sampling. Inclusion criteria were middle‐aged (40–65 y) endurance athletes living in Canada and participating in ≥ 8 h of moderate‐vigorous aerobic training per week (e.g., long distance athletes such as marathon runners, ultra runners, triathletes, adventure racers, etc.). Exclusion criteria were any athletes that self‐disclosed their physical activity volume and/or dietary intake changed due to COVID‐19 induced restrictions that occurred in March of 2020. Additional exclusion criteria were women who were pregnant or breastfeeding, or anyone with a digestive disorder (e.g., celiac, irritable bowel syndrome, Crohn's, etc.). Informed consent and eligibility criteria were obtained using an online survey platform [Qualtrics] explaining the study with the following statement: “By clicking ‘I agree’ below, you acknowledge that you have read and understand the description provided, and as such consent to participate in this research study.” The study procedures were approved by the Research Ethics Board at the University of Regina (REB #2019‐212).

### 2.2 Online Survey

The online survey [Qualtrics] was used to collect demographic information (age, sex/gender, location of residence, self‐reported height and body mass), medical history (menopausal status for females, current or history of disease, current or history of smoking, medication use), current physical activity levels (International Physical Activity Questionnaire Short Form [IPAQ‐SF]) [23], and whether physical dietary intake changed due to COVID‐19‐induced restrictions: “Has the current COVID‐19‐induced restrictions changed the way you eat?” (with “how” and “why” follow‐up questions).

### 2.3 Food Intake Records and Nutrient Analyses

Eligible participants identified from the online survey were contacted and provided with instructions to complete a three‐day food intake record (FIR) on two non‐consecutive weekdays and one weekend day. Details including type of food and drink consumed, method of preparation, portion size consumed, and vitamin and mineral supplements were recorded. Written and verbal instructions on how to complete the FIR were provided via email and an unlisted YouTube video, respectively. If participants did not have access to a food scale, additional information on estimating portion size using a catalogue of pictures of individual food portions was provided. FIRs were reviewed by a trained research assistant [author O.M.] for clarity and detail; participants were contacted via email if additional information was required.

All food, beverage, and supplemented food and beverage items (e.g., bars or drinks with added micronutrients, protein powders, etc.) were entered by O.M. into Cronometer (v2.18.6), a freely available dietary assessment software that allows for analysis of up to 82 nutrients with information sourced from verified databases including the Canadian Nutrient File and the United States Department of Agriculture National Nutrient Database. Of note, vitamin and mineral supplements were not entered into the nutrient analysis software, and were assessed separately. A recent publication assessing the reliability and validity of Cronometer describes in more detail the standard operating procedure used by O.M. to ensure precision and accuracy of food and beverage input [24].

Data were exported to Excel where the 3‐day average intake for each participant was calculated for macronutrients and micronutrients. Estimated energy requirement (EER, using the Mifflin‐St Jeor equation and a physical activity coefficient of moderately active [1.55], very active [1.725], or extremely active [1.9]) was calculated [25] and compared to total energy intake (kcal) from the Cronometer output as “meeting,” or “not meeting” recommended intake. Macronutrient intakes were compared to dietary reference intakes (DRIs): recommended dietary allowance (RDA) (carbohydrate and protein), acceptable macronutrient distribution ranges (AMDR) (% of total intake for carbohydrate, protein, fat) [26], and sport nutrition recommendations (g/kg/day for carbohydrate and protein) [9] as “meeting,” “not meeting,” or “exceeding” recommended intakes. Water, fiber, omega‐3, and omega‐6 (mL/day or g/day) were compared to DRI Adequate Intake (AI), and micronutrient intakes were compared to DRI recommendations (RDA or AI), as “meeting,” “not meeting,” or “exceeding” recommended intakes [26].

### 2.4 Statistical Analyses

Descriptive statistics were computed for demographics, medical history, and physical activity as mean ± standard deviation, median, and range, or as count (*n*, %). Average daily macronutrient and micronutrient intake parameters were calculated as mean ± standard deviation for the total cohort as well as for males and females separately; unpaired sample *t*‐tests were used to compare nutrient intake between males and females.

Fisher's Exact Test was used to compare the proportion of male and female endurance athletes that were meeting and exceeding the energy, macronutrient, and micronutrient recommendations. Shortfall nutrients were defined as < 75% of the total cohort meeting the recommended intakes (DRI RDA or AI), loosely based off similar previous work [27]. When inadequate micronutrient intake was observed (i.e., shortfall nutrients), vitamin and mineral supplements were considered, and Fisher's Exact Test was used to compare the proportion of male and female endurance athletes that were meeting the recommendations (i.e., food intake plus vitamin and mineral supplement(s)). Data were analyzed using IBM SPSS version 28.0.1.0 (IBM Inc., Armonk, NY), and figures were generated using GraphPad Prism version 9.0 (GraphPad Software, La Jolla, CA).

## 3 Results

Of the 55 participants who initiated the study (i.e., completed the online Qualtrics survey), 11 were omitted due to not living in Canada (*n* = *2*), stating that their diet changed due to COVID‐19‐induced restrictions (*n* = *2*), and not completing the FIR (*n* = *7*); data from a total of 44 Canadian middle‐aged endurance athletes were used for the present study (28 M/16 F). Of note, the sample size is similar to previous dietary intake studies (from 10 to 80 participants) [21]. Of the 16 female participants, five were premenopausal, two were perimenopausal, and nine were postmenopausal; data were pooled as no differences were found between menopausal states for any nutrient outcomes. Athletes were loosely classified in accordance with De Pawu guidelines based on training volume, frequency, and years of experience [28]. Performance Level 3 (PL3) were athletes that trained for ≥ 5 h/wk on ≥ 3 days/wk (*n* = 25; 56%); Performance Level 4 (PL4) were athletes that trained for ≥ 10 h/wk on ≥ 3 days/wk for ≥ 3 years (*n* = 13; 30%); and Performance Level 5 (PL5) were athletes that trained for ≥ 10 h/wk on ≥ 5 days/wk for ≥ 5 years (*n* = 6; 14%) [28]. Athletes in the present study trained 10.5 ± 3.9 h per week for 11.2 ± 8.6 years. Participant characteristics can be found in Table 1.

**Table 1:** Participant characteristics.

Parameter	Total (*n* = 44)	Male (*n* = 28)	Female (*n* = 16)
Age, y	50.4 ± 7.2	50.2 ± 8.3	50.7 ± 5.5
Height, m	1.74 ± 0.11	1.82 ± 0.89	1.64 ± 0.48
Body Mass, kg	72.3 ± 16.3	81.1 ± 13.7	59.2 ± 10.4
BMI, kg/m^2^	23.6 ± 3.2	24.7 ± 2.8	22.0 ± 3.2
MVPA, min/week	627.0 ± 232.0	634.0 ± 266.7	615.9 ± 170.
Time exercising at this volume, years	11.2 ± 8.6	12.3 ± 8.3	9.5 ± 9.2
***Performance Level***			
PL 3	25 (56)	15 (53)	10 (63)
PL 4	13 (30)	8 (29)	5 (31)
PL 5	6 (14)	5 (18)	1 (6)
***Location of Residence***			
Saskatchewan	26 (59)	15 (54)	11 (69)
Ontario	15 (34)	11 (39)	4 (25)
Quebec	2 (5)	2 (7)	‐‐
British Columbia	1 (2)	‐‐	1 (6)
***Menopause Status***			
Pre‐Menopause	5 (31.3)	‐‐	5 (31.3)
Peri‐Menopause	2 (12.5)	‐‐	2 (12.5)
Post‐Menopause	9 (56.3)	‐‐	9 (56.3)
***Smoking Status***			
Current	0 (0)	0 (0)	0 (0)
Past	11 (25)	4 (14)	4 (25)
Never	33 (75)	24 (86)	12 (75)
***Prescription Medications***			
Yes	12 (27)	8 (29)	4 (25)
No	32 (73)	20 (71)	12 (75)

*Notes:* Data are presented as mean ± standard deviation or count as *n* (%). Regular aerobic exercise activities included: running, biking, swimming, paddling, hockey, soccer, skiing.

PL = performance level: PL3 were athletes that trained for ≥ 5 h/wk on ≥ 3 days/wk; PL4 were athletes that trained for ≥ 10 h/wk on ≥ 3 days/wk for ≥ 3 years; and PL5 were athletes that trained for ≥ 10 h/wk on ≥ 5 days/wk for ≥ 5 years.

Current prescription medications to help treat the following: depression (x2), anxiety (x1), attention deficit hyperactivity disorder (x1), hypothyroid (x2), glaucoma (x1), high blood pressure (x1), asthma (x1), cholesterol (x2), prostate (x2).

Abbreviations: BMI = body mass index (note: height and body mass were self‐reported), MVPA = moderate to vigorous physical activity.

Normality of distribution for nutrients was examined prior to analysis using a combination of histograms and the Shapiro‐Wilks test; all data were normally distributed. Average daily macronutrient and micronutrient intake of the total cohort as well as for males and females can be found in Tables 2 and 3, respectively, which also outlines DRI, AMDR, and/or sports nutrition recommended intakes. When examining sex differences, absolute values of total energy (kcal), carbohydrate (g), protein (g), and fat (g) intake were higher among males compared to females (*p* < 0.01), however when normalized to body mass (g/kg) these differences disappeared. Sugar intake (g/day) was higher in males versus females (*p* < 0.01), however when calculated as a percent of total intake (E%), this difference disappeared. Fiber intake (g/day) was higher in males versus females (*p* < 0.01); however, the proportion of males and females meeting the DRI AI recommendations was similar (*p* = 1.0). Of note, post hoc power calculations conducted using Fisher's exact test, based on the sample size and Cohen's *w* effect size at an alpha level of 0.05 (two‐tailed), demonstrated that the sex‐based comparisons were adequately powered.

**Table 2:** Average daily macronutrient intake.

Macronutrient	Total (*n*=44)	Male (*n*=28)	Female (*n*=16)	DRI: M, F or AMDR	Sport Nutrition Recommendations
*TOTAL kcal	2541 ± 675	2857 ± 574	1987 ± 443		a3119 ± 300; 2220 ± 211
kcal/kg	36 ± 9	36 ± 9	34 ± 10		
Water, mL	3438 ± 1467	3534 ± 1560	3269 ± 1318	*3700; 2700*	Replace 1.5x what was lost
CHO (4 kcal/g)					
*g	279 ± 109	318 ± 115	211 ± 50	≥ 130, ≥ 130	
g/kg	3.9 ± 1.5	4.1 ± 1.7	3.7 ± 1.2		5–10 g/kg/d [14]
E%	44 ± 10	44 ± 11	43 ± 9	45–65% of total intake	50–65% of total intake [14]
*Sugars, g	108 ± 57	123 ± 64	82 ± 31		
E%	17 ± 6	17 ± 7	16 ± 5		
*Fibre, g	37 ± 13	42 ± 14	29 ± 7	*38–30, 25–21*	
PRO (4 kcal/g)					
*g	119 ± 37	135 ± 33	92 ± 28	56, 46	
g/kg	1.7 ± 0.4	1.7 ± 0.4	1.6 ± 0.5	0.8, 0.8	1.2–2.0 g/kg/d [14]
E%	19 ± 4	19 ± 4	19 ± 5	10–35% of total intake	
FAT (9 kcal/g)					
*g	108 ± 37	116 ± 34	86 ± 33		
g/kg	1.5 ± 0.5	1.5 ± 0.4	1.5 ± 0.6		
E%	37 ± 9	37 ± 9	38 ± 7	20–35% of total intake	Do not restrict to < 20%
*MUFA, g	34 ± 15	39 ± 15	24 ± 12		
PUFA n‐3, g	2.3 ± 1.8	2.6 ± 2.1	1.7 ± 0.8	*1.6, 1.1*	
E%	0.8 ± 0.6	0.9 ± 0.7	0.8 ± 0.3	0.6–1.2% of total intake	
PUFA n‐6, g	16 ± 9	18 ± 9	14 ± 9	*17–14, 12–11*	
E%	6 ± 3	6 ± 3	6 ± 3	5–10% of total intake	
Saturated, g	32 ± 15	34 ± 14	27 ± 15	Minimize intake	
E%	11 ± 4	11 ± 4	12 ± 4	< 10% of total intake	
Trans, g	3.5 ± 6.1	3.2 ± 5.3	4.1 ± 7.5	Minimize intake	
Caffeine, mg	212 ± 195	221 ± 230	197 ± 117		

*Notes:* Data are presented as mean ± standard deviation. Protein intake includes protein powder (*n* = 10M/7F reported intake of protein powder). DRIs are presented as Recommended Dietary Allowance or *Adequate Intake (in italics)* for males, females in 31–50y and 51–70y age groups.

Abbreviations: AMDR = acceptable macronutrient distribution range; CHO = carbohydrates; DRI = dietary reference intakes; E% = percent energy of total intake; F = female; M = male; MUFA = monounsaturated fat; PRO = proteins; PUFA = polyunsaturated fat. *p < 0.01 M vs. F.

^a^ Estimated Energy Requirement (kcal/day) for adults 19–78 years calculated using the Mifflin‐St Jeor equation and a physical activity coefficient of *moderately active* (1.55), *very active* (1.725), or *extremely active* (1.9).

**Table 3:** Average daily micronutrient intake.

Micronutrient	Total (*n* = 44)	Male (*n* = 28)	Female (*n* = 16)	DRI: M, F ∣UL
Fat‐soluble vitamins				
Vitamin A, IU	4928 ± 3617	4170 ± 3669	4912 ± 2416	3000, 2333 ∣10,000
Vitamin D, IU	221 ± 181	247 ± 88	176 ± 218	600, 600 ∣4000
*Vitamin E, mg	13 ± 8	15 ± 8	10 ± 4	15, 15 ∣1000
Vitamin K, µg	228 ± 192	211 ± 158	256 ± 246	*120, 90* ∣ND
Water‐soluble vitamins				
*Vitamin B1, mg	1.6 ± 0.6	1.8 ± 0.6	1.1 ± 0.3	1.2, 1.1 ∣ND
*Vitamin B2, mg	2.4 ± 0.9	2.6 ± 0.9	1.9 ± 0.8	1.3, 1.1 ∣ND
*Vitamin B3, mg	28 ± 11	33 ± 11	22 ± 7	16, 14 ∣35
*Vitamin B5, mg	7 ± 3	8 ± 3	5 ± 2	*5, 5* ∣ND
*Vitamin B6, mg	2.4 ± 0.9	2.7 ± 0.9	1.9 ± 0.6	1.3–1.7, 1.3–1.5 ∣100
*Vitamin B9, µg	450 ± 168	499 ± 182	365 ± 92	400, 400 ∣1000
*Vitamin B12, µg	4.8 ± 2.7	5.6 ± 2.8	3.4 ± 17	2.4, 2.4 ∣ND
Vitamin C, mg	138 ± 80	147 ± 89	122 ± 63	90, 75 ∣2000
Major minerals				
Calcium, mg	1141 ± 431	1196 ± 405	1044 ± 470	1000, 1000‐1200 ∣2500‐2000
*Magnesium, mg	454 ± 154	503 ± 162	368 ± 92	420, 320 ∣^a^350
*Phosphorous, mg	1503 ± 462	1689 ± 415	1179 ± 353	700, 700 ∣4000
*Potassium, mg	3683 ± 984	4051 ± 953	3039 ± 670	4700, 4700 ∣ND
Sodium, mg	3102 ± 1509	3270 ± 1372	2809 ± 1732	1500–1300, 1500–1300 ∣^b^2300
Trace minerals				
*Copper, mg	1.8 ± 0.6	2.0 ± 0.5	1.5 ± 0.7	0.9, 0.9 ∣10.0
*Iron, mg	18 ± 5	19 ± 5	15 ± 3	8, 18‐8 ∣45
*Manganese, mg	5.0 ± 2.2	5.5 ± 2.2	3.8 ± 1.8	*2.3, 1.8* ∣11
Selenium, µg	148 ± 114	161 ± 67	124 ± 168	55, 55 ∣400
*Zinc, mg	14 ± 6	16 ± 6	10 ± 4	11, 8 ∣40

*Note:* Data are presented as mean ± standard deviation. Dietary reference intakes (DRI) presented as Recommended Dietary Allowance or *Adequate Intake (in italics)* for males, females in 31–50 y and 51–70 y age groups, followed by the tolerable upper limit levels (UL; ND = not determined). *p < 0.01 M versus F absolute nutrient intake

^a^ UL for magnesium represents intake from a pharmacological agent only and does not include intake from food and water (i.e., % exceeding DRI UL is from supplements).

^b^ UL for sodium was replaced by the chronic disease risk reduction (CDRR) intake value in 2019, the level above which a reduction in intake is expected to reduce chronic disease risk.

### 3.1 Proportion of Cohort Meeting Recommended Intakes

#### 3.1.1 Total Energy, Water, Fiber, Macronutrients

**Figure 1:**
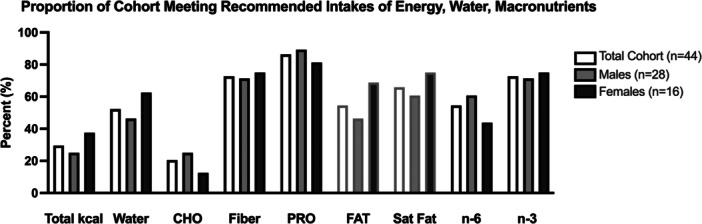
Black boarder = % meeting recommended intakes; grey boarder = % *exceeding* recommended intakes. Total kcal compared to recommended intake for *moderately active, very active* or *extremely active* males and females (see text for equation). Water, fiber, omega‐6 (n‐6), and omega‐3 (n‐3) intakes compared to Dietary Reference Intake (DRI) Adequate Intake (AI) for males and females in 31–50 y and 51–70 y age groups (mL or g/day). Carbohydrate (CHO; 5–10 g/kg/day) and protein (PRO; 1.2‐2.0 g/kg/day) intakes compared to sports nutrition guidelines for endurance athletes. Fat and saturated fat (Sat Fat) intake compared to Acceptable Macronutrient Distribution Range (AMDR, % of total intake) and represented as proportion of cohort *exceeding* recommended intakes. No differences between males and females.

The proportion of the total cohort as well as the proportion of males and females meeting recommended intakes for total energy, water, and macronutrients (including fibre) are represented in Figure 1. Only 29.5% (*n* = 13) of the total cohort met the recommendations for total energy intake (i.e., calculated EER for *moderately active, very active*, or *extremely active* [29]) (M 25.0% [*n* = 7] v F 37.5% [*n* = 6]; *p* = 0.250), and only half of the participants (*n* = 23 [52.3%]) met the DRI AI for water (M 46.4% [*n* = 13] v F 62.5% [*n* = 10]; *p* = 0.360).

Only 27.3% (*n* = 12) of the total cohort fell within range of the sport nutrition guidelines for CHO of 50%–65% of total kcal intake (M 28.6% [*n* = 8] vs. F 25.0% [*n* = 4]; *p* = 0.544), and only 20.5% (*n* = 9) fell within range of the sport nutrition guidelines for CHO of 5–10 g/kg/day (M 25.0% [*n* = 7] v F 12.5% [*n* = 2]; *p* = 0.450). However, 72.7% (*n* = 32) met the DRI AI for fiber (M 71.4% [*n* = 20] v. F 75.0% [*n* = 12]; p = 1.0). The entire cohort met the AMDR for PRO of 10%–35% of total kcal intake, and nearly all (86.4% [*n* = 38]) met the sport nutrition guidelines for PRO of 1.2–2.0 g/kg/day (M 89.3% [*n* = 25] v F 81.3% [*n* = 13]; *p* = 0.652). Regarding fat intake, 54.5% (*n* = 24) of the cohort *exceeded* the AMDR upper end of 35% fat of total kcal intake (M 46.4% [*n* = 13], F 68.8 [*n* = 11]; *p* = 0.213), and 65.9% (*n* = 29) *exceeded* the recommended intake for saturated fat of < 10% (M 60.7% [*n* = 17] v F 75.0% [*n* = 12]). Of the total cohort, 54.5% (*n* = 24) and 72.7% (*n* = 32) met the DRI AI for omega‐6 (M 60.7% [*n* = 17] v F 43.8% [*n* = 7]; *p* = 0.352) and omega‐3 (M 71.4% [*n* = 20] v F 75.0% [*n* = 12]; p = 1.0) intake (g/day), respectively. Of note, the ratio of omega‐6 to omega‐3 was 9.5 ± 6.9:1 (M 9.8 ± 8.0:1 v F 8.9 ± 4.4:1; *p* = 0.65).

#### 3.1.2 Shortfall Nutrients and Supplements; and Sodium

**Figure 2:**
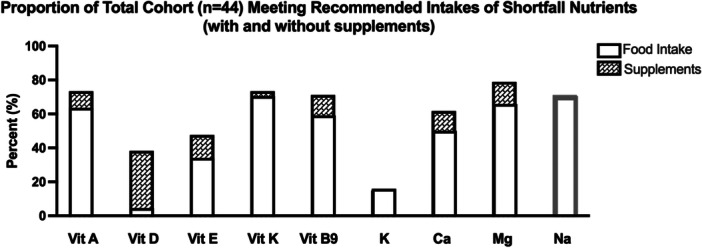
Shortfall micronutrients and sodium. Shortfall nutrients are defined as less than 75% of the total cohort meeting recommended intakes. Black border = % meeting recommended intakes; grey border = % *exceeding* recommended intakes. Fat‐soluble vitamins (A, D, E, K), water‐soluble vitamin B9, and minerals (potassium, K; calcium, Ca; magnesium, Mg; sodium, Na) compared to DRI Recommended Dietary Allowance (RDA) or Adequate Intake (AI) for males and females in 31–50 y and 51–70 y age groups (IU, mg, or µg/day). White bars represent total cohort micronutrient intake from whole foods only; patterned bars represent micronutrient intake from whole foods plus supplements.

Less than 75% of the total cohort met the recommended intakes (DRI RDA or AI) for: vitamin A (63.6%, *n* = 28), vitamin D (4.5%, *n* = 2), vitamin E (34.1%, *n* = 15), vitamin K (70.5%, *n* = 31), vitamin B9 (folate, 59.1%, *n* = 26), potassium (15.9%, *n* = 7), calcium (50%, *n* = 22), and magnesium (65.9%, *n* = 29). For sodium, 70.5% of the total cohort *exceeded* the DRI chronic disease risk reduction (CDRR) (*n* = 31) (Figure 2). Twenty‐six participants (59.1%) reported consuming vitamin and mineral supplements; the most common were multivitamins (48%), antioxidants (48%), vitamin D (36%), and magnesium (36%). Of note, most multivitamins contain fat‐soluble and water‐soluble vitamins as well as a variety of minerals (including calcium and magnesium) [30], and therefore multivitamins were considered towards “total intake” of these micronutrients. When adding vitamin and mineral supplementation to food intake (i.e., “total intake”), the proportion of the total cohort meeting the recommended intakes (DRI RDA or AI) increased for: vitamin A (63.6% to 72.7%), vitamin D (4.5% to 38.6%), vitamin E (34.1% to 47.7%), vitamin K (70.5% to 72.7%), vitamin B9 (59.1% to 70.5%), calcium (50.0% to 61.4%), and magnesium (65.9% to 78.0%) (Figure 2).

**Figure 3:**
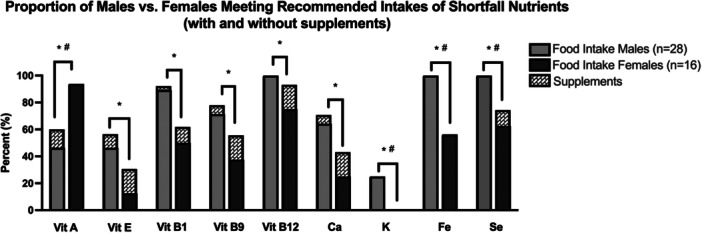
Males versus females for shortfall micronutrients. Shortfall nutrients are defined as less than 75% of males or females meeting recommended intakes. Vitamins (A, E, B1, B9, B12) and minerals (Ca, Calcium; K, Potassium; Fe, Iron; Se, Selenium) compared to DRI Recommended Dietary Allowance (RDA) or Adequate Intake (AI) for males and females in 31–50 y and 51–70 y age groups (IU, mg, or µg/day). Light grey and dark grey bars represent micronutrient intake from whole foods in males and females, respectively; *p < 0.05 M versus F meeting recommended intakes from whole foods only. Patterned bars represent micronutrient intake from whole foods plus supplements; #*p* < 0.05 M versus F for whole foods plus supplement (i.e., if significance remained between males and females after adding supplement intake).

The proportion of males compared to females meeting the recommended intakes (DRI RDA or AI) of shortfall nutrients are represented in Figure 3. Males were at risk compared to females for not meeting the recommended intake for vitamin A (M 46.4% [*n* = 13] v F 93.8% [*n* = 15], *p* = 0.003). Females were at risk compared to males for not meeting the recommended intakes for: vitamin E (M 46.4% [*n* = 13] v F 12.5% [*n* = 2], *p* = 0.045), vitamin B1 (thiamin, M 89.3% [*n* = 25] v F 50% [*n* = 8], *p* = 0.009), vitamin B9 (folate, M 71.4% [*n* = 20] v F 37.5% [*n* = 6], *p* = 0.054), vitamin B12 (cobalamin, 100.0% [*n* = 28] v F 75.0% [*n* = 12], *p* = 0.013), calcium (M 64.3% [*n* = 18] v F 25.0% [*n* = 4], *p* = 0.027), potassium (M 25.0% [*n* = 11] v F 0.0% [*n* = 0], *p* = 0.037), iron (100% [*n* = 28] v F 56.3% [*n* = 9], *p* < 0.001) and selenium (100% [*n* = 28] v F 62.5% [*n* = 10], *p* = 0.001). The difference between males and females meeting recommended intakes of vitamin E, vitamin B1, vitamin B9, vitamin B12, and calcium disappeared when adding vitamin and mineral supplements; however, the difference between sexes remained after adding vitamin and mineral supplements to food intake for vitamin A, potassium, iron, and selenium. Of note, while there were *n* = 2 females taking iron supplements, these two athletes were already meeting recommended intakes from whole foods (Figure 3).

#### 3.1.3 Other Micronutrients

Over 75% of the total cohort met the recommended intakes (DRI RDA or AI) for: vitamin B1 (thiamin) 75.0% (*n* = 33); vitamin B2 (riboflavin) 93.2% (*n* = 41); vitamin B3 (niacin) 88.6% (*n* = 39); vitamin B5 (pantothenic acid) 77.3% (*n* = 34); vitamin B6 (pyridoxine) 81.8% (*n* = 36); vitamin B12 (cobalamin) 90.9% (*n* = 40); vitamin C (ascorbic acid) 79.5% (*n* = 35); phosphorous 100% (*n* = 44); copper 95.5% (*n* = 42), iron 84.1% (*n* = 37), manganese 93.2% (*n* = 41), selenium 86.4 (*n* = 38), and zinc 77.3% (*n* = 34).

## 4 Discussion

The present study comprehensively analyzed macronutrient and micronutrient intakes among non‐elite middle‐aged endurance athletes, with a focus on sex differences. Several key findings emerged from this study, all of which have practical implications for understanding and improving the nutritional status of middle‐aged male and female endurance athletes: (1) most athletes did not meet recommended intakes of total kcal or CHO, and exceeded recommendations for FAT, with no differences between sexes; (2) most athletes did not meet recommended intakes of several micronutrients, with females being at greater risk than males, and (3) over half the cohort consumed supplements that increased total intakes of several shortfall nutrients. Of note, the overall dietary pattern findings align with intakes among the Canadian general population > 19 years of age [31, 32].

### 4.1 Total Energy, Water, Macronutrients

Findings of inadequate total kcal and water intake in the present cohort of both male and female middle‐aged endurance athletes align with several previous studies among collegiate and young elite endurance athletes [33, 34, 35, 36] and a more recent study among non‐elite endurance athletes (age: 34.9 ± 12.9 y) [10]. Consuming adequate total kcal and water are important for middle‐aged endurance athletes not only for optimal athletic performance, but also for muscle maintenance and recovery, bone health, hormonal regulation, immune function, hydration, and overall healthy aging [14].

Similar to previous literature among young high‐performance [19, 35, 37] and non‐elite endurance athletes [38, 39], middle‐aged endurance athletes in the present study did not meet the recommended intake of CHO [9]. Since CHO are essential for energy, recovery, and overall health, inadequate intake can be detrimental particularly among middle‐aged endurance athletes due to their high energy needs and age‐related physiological changes [14]. Conversely, and similar to previous findings [10, 19], 54.5% (*n* = 24) of the total cohort *exceeded* the AMDR for total FAT intake (20%–35% of total kcal intake). Of important consideration is the increased popularity of low CHO high FAT, or ketogenic, diets among athletes [40]. While athletes in the present study were not asked if they were intentionally adhering to a ketogenic diet, *n* = 4 (2 M/2 F) were at or below the DRI RDA for CHO (< 130 g/day), including *n* = 1 male at the definition of a ketogenic diet [approximately 10% CHO, 20% PRO and 70% FAT] [41]. While some evidence supports the use of these diets for enhanced performance [42], much of the literature suggests no change or impaired performance [40, 43, 44]. Regarding saturated fat, over 65% of the athletes *exceeded* recommended intakes (< 10% of total kcal intake), similar to previous findings [37, 45]. Increased saturated fat intake is associated with increased risk of obesity and cardiovascular disease [46, 47].

Some encouraging findings from the present cohort were related to dietary fiber and essential fatty acids intake. That is, most athletes met the DRI AI for daily fiber intake (M 71.4% and F 75.0%), a critical nutrient for gastrointestinal health including gut microbiota composition and function [48], and microbial byproducts (e.g. short chain fatty acids) that can subsequently impact athletic performance [49, 50]. Further, contrary to previous work reporting low adherence to omega‐3 intake [10, 19], > 75% of the present cohort met the recommended intakes in the present study. Sufficient omega‐3 intake decreases exercise‐induced inflammatory markers that promotes health and performance. The balance of omega‐6 to omega‐3 is important to optimize performance and recovery; typical Western diets have excess omega‐6 and are deficient in omega‐3 resulting in an increased ratio of omega‐6 to omega‐3 that promotes an inflammatory environment [51]. The ratio in the present study was 9.5 ± 6.9:1, higher than the ideal ratio of 4:1, but less than the reported typical Western diet ratio of 15:1–16.7:1 [51].

One hundred percent of the present cohort met the AMDR for PRO (*n* = 44), and 86.4% (*n* = 38) met the sport nutrition guidelines of 1.2–2.0 g/kg/day [9] with no difference between sexes; these findings are particularly encouraging considering age‐related decreases in muscle mass [14]. Previous studies have similarly reported sufficient PRO intakes among endurance athletes, but further reported females do not meet PRO intake guidelines compared to males [10, 19]. It has been recommended that masters endurance athletes increase their PRO intake to 1.6 g/kg/day to promote optimal post‐exercise recovery and prevent muscle mass and bone density losses associated with the aging process [14]; in the present cohort 64.3% (*n* = 18) males and 43.8% (*n* = 7) females met the 1.6 g PRO/kg/day intake guideline (*p* = 0.220). Of note, intake of leucine in the present cohort (M 99.3 ± 27.7 and F 92.7 ± 47.6 mg/kg/day) was above the 55 mg/kg/day recommended levels for those engaging in intensive training [14, 52] for most of the present cohort (M 89.3% [*n* = 25] v F 81.3% [*n* = 13]; *p* = 0.652). This is an important finding as leucine is an essential amino acid that cannot be synthesized by the human body, and is responsible for muscle growth, strength and enhanced athletic performance [14, 52, 53].

### 4.2 Micronutrients—Vitamins and Minerals

Numerous previous studies have reported findings of inadequate intakes of most micronutrients among young elite athletes [19, 37, 54, 55, 56, 57]. A recent systematic review assessing dietary intake among master's athletes (> 35 y of age) showed eight of the 26 included studies reported on five key micronutrients: they found higher iron, calcium, and sodium intakes among the athletes compared to the general Australian population [21]. When comparing intakes from the present study to average intakes of middle‐aged Canadians [32], similar findings of higher iron, calcium, and sodium intakes among the athletes were observed. However, when assessing the proportion of athletes in the present study meeting the DRIs (RDA or AI), the athletes were not meeting the micronutrient intake recommendations for iron (females only) or calcium, as well as additional micronutrients of vitamins A, D, E, K, B9, potassium, or magnesium. As athletes age, prioritizing recovery, muscle preservation, and bone health become more important; fat‐soluble vitamins, vitamin B9, calcium, potassium and magnesium work together to enhance endurance, prevent injury, and support performance. Promisingly, several athletes in the present cohort reported taking vitamin D and calcium supplements (and/or multivitamins that typically contain these micronutrients), particularly important in this Canadian cohort who do not get enough vitamin D from sunlight between October and March [58]. Regarding sodium, most of the participants in the present study exceeded the recommended intake from their diet (DRI CDRR 2300 mg/day). Of note, the CDRR (formerly UL) was derived for the general population with the primary goal of reducing chronic disease risk and do not account for the higher sodium losses incurred during prolonged endurance exercise. While there are no specific sodium intake guidelines for athletes, a recent review suggested sodium intake should be individualized based on sweat rate, sweat sodium concentration, environment, and the athlete's health history [59]. Importantly, overconsumption of sodium among athletes does not confer performance benefits [60] and may pose health risks [61].

Regarding sex differences, males were at a greater risk compared to females for not meeting the recommended intake of Vitamin A, contrary to a recent study reporting the opposite among younger non‐elite endurance athletes (average age 34.9 y) [10]. In the present cohort, females were at a greater risk compared to males for not meeting the recommended intakes of vitamin E, vitamin B1, vitamin B9, vitamin B12, calcium, potassium, iron, and selenium. Females have different nutrient needs due to fluctuations in sex hormones, substrate reliance, and increased energy demand during exercise, and females may have deficiencies in certain nutrients due to factors like menstrual losses, bioavailability, and dietary habits [15, 62, 63, 64]. Female athletes who to not consume enough B vitamins or iron may influence their ability to convert macronutrients into energy during exercise as well as increase the risk of anemia, increasing fatigue and decreasing endurance [55, 65]. Female athletes reported taking B vitamins (31.3%, *n* = 5), iron (12.5%, *n* = 2), and multivitamin (18.8%, *n* = 3) supplements; the two female athletes that took iron supplements were already meeting the DRIs. As female athletes transition through perimenopause and post menopause, hormonal changes impact bone density, muscle mass, energy metabolism, and cardiovascular health [66]. Unfortunately, the sample size of females in the present study was too small to evaluate differences in nutrient intake by menopause status, a research area of great importance for future research.

### 4.3 Strengths and Limitations

A strength of the present study was the comprehensive nature of evaluating both macronutrients and micronutrients among the under‐represented cohort of middle‐aged endurance athletes. A methodological strength was the utilization of a three‐day non‐consecutive (two weekdays and one weekend day) FIR; while this method of nutrient intake data collection has limitations including potential for reactivity (i.e., changing eating habits) and underreporting, its validity is higher than food frequency questionnaires [67]. Further, participants were given instructions reiterating the importance of reporting accurate intake quantities, and participants were contacted when additional information on a food entry was required. The use of a reliable and valid nutrient assessment software–Cronometer [24] – is another methodological strength. Limitations include the relatively small sample size and cross‐sectional design that may not reflect long‐term consumption patterns. As nutrient recommendations are typically expressed relative to body mass, reliance on self‐reported body mass represents a limitation. Further, athletes were not asked to record training volume or intensity on the days they recorded food intake, which could influence dietary needs and intake patterns. Lastly, protein intake across the day was not considered; research suggests distributed protein intake leads to greater lean‐mass gains and recovery from exercise for master athletes [68].

## 5 Conclusions

Regular exercise training can help counteract many age‐related declines in body composition, muscle function, metabolic efficiency, etc.; however, exercise must be paired with adequate nutrient intake to be effective. Middle‐aged athletes require diets that not only support physical performance but also promote healthy aging. Findings from the current study suggest middle‐aged endurance athletes are not meeting recommended intakes of total kal and CHO as well as many micronutrients, and are exceeding recommended intakes of saturated FAT and sodium. Of note, these overall dietary patterns are similar to the general population > 19 years of age in Canada [31, 32]. Improving adherence to recommended intakes may help these athletes achieve optimal performance, taking into consideration sex‐ and age‐related physiological changes. Further, findings may assist dietitians, physicians, and coaches in properly counselling middle‐aged endurance athletes regarding their dietary intake. Future directions should investigate longitudinal dietary intake among middle‐aged endurance athletes, as well as dietary intake based on menopausal stage.

## Author Contributions

**B. Ghomeshi:** data curation (lead), formal analysis (supporting), writing – original draft (supporting). **L. McPhee:** data curation (supporting), writing – review & editing (supporting). **O. Morello** formal analysis (lead), writing – review & editing (supporting). **M. Ismail:** data curation (supporting), writing – review & editing (supporting). **M. Kebbe:** methodology (supporting), writing – review & editing (supporting). **J. O. Totosy de Zepetnek:** conceptualization (lead), data curation (supporting), formal analysis (lead), methodology (lead), project administration (lead), resources (lead), supervision (lead), visualization (lead), writing – original draft (lead).

## Funding

The authors received no specific funding for this work.

## Ethics Statement

The study procedures were approved by the Research Ethics Board at the University of Regina (REB #2019‐212).

## Conflicts of Interest

The authors declare no conflicts of interest.

## Data Availability

The data underlying this article will be shared on reasonable request to the corresponding author.
